# Applicability and feasibility of robot-assisted cystectomy and intracorporeal urinary diversion in a patient with right renal pelvic ectopia

**DOI:** 10.1590/S1677-5538.IBJU.2023.0608

**Published:** 2024-03-18

**Authors:** Stefano Puliatti, Stefania Ferretti, Natali Rodriguez Peñaranda, Ahmed Eissa, Marco Ticonosco, Andrea De Faveri, Cosimo De Carne, Pawel Wisz, Riccardo Ferrari, Greta Tosi, Filippo Annino, Giampaolo Bianchi, Salvatore Micali

**Affiliations:** 1 Azienda Ospedaliero-Universitaria di Modena Department of Urology Italy Department of Urology, Azienda Ospedaliero-Universitaria di Modena, Italy;; 2 University of Modena and Reggio Emilia Modena Italy University of Modena and Reggio Emilia, Modena, Italy;; 3 Tanta University Faculty of Medicine Urology Department Tanta Egypt Urology Department, Faculty of Medicine, Tanta University, Tanta, Egypt;; 4 Orsi Academy Melle Belgium Orsi Academy, Melle, Belgium;; 5 Ospedale San Donato Unit of Urology Arezzo Italy Unit of Urology, Ospedale San Donato, Arezzo, Italy

## Abstract

**Background:**

The ectopic pelvic kidney, a common renal anomaly, is often smaller and malformed, with a shorter and sometimes tortuous ureter (1). Muscle-invasive bladder cancer (MIBC), constituting 15-25% of bladder cancer cases (2), mandates radical cystectomy with a 50% 5-year survival rate (2). Despite the growing use of robot-assisted radical cystectomy (RARC) (3, 4), there is limited data on its application in ectopic kidneys. Only one RARC case has been reported (5), in contrast to numerous open radical cystectomies (1, 6) involving an ectopic kidney.

**Patient and methods:**

After being diagnosed with T2 high-grade urothelial carcinoma, the 66-year-old patient, previously treated with multiple transurethral resections and adjuvant BCG therapy, received neoadjuvant chemotherapy. Preoperative staging CT revealed a 2.6 x 2.2 cm bladder neoformation and an ectopic right pelvic kidney.

**Results:**

Using the da Vinci Surgical System, radical cystectomy with ileal conduit (sec Wallace II) and lymphadenectomy were performed. During the demolition phase, the shorter right ureter was dissected with care to avoid damage to the renal pedicle. The reconstructive phase included intracorporeal urinary diversion (ICUD) and uretero-ileal anastomosis, facilitated by the favorable position of the kidney. The 8-hour console surgery resulted in minimal blood loss. Discharged on day 16 due to COVID-19, the patient exhibited positive outcomes. A 2-month CT follow-up revealed no cancer recurrence, metastasis, hydronephrosis, and complete regression of the lymphocele. Imaging follow-up continues without postoperative adjuvant chemotherapy.

**Conclusion:**

Robotic surgery with intracorporeal urinary diversion holds potential for right-sided pelvic kidney cases, but additional studies are necessary for validation.

Available at: http://www.intbrazjurol.com.br/video-section/20230608_Puliatti_et_al

**Int Braz J Urol. 2024; 50 (Video #3): 227-8**
